# Effect of Head Covering on Phototherapy-Induced Hypocalcaemia in Icterus Newborns; A Randomized Controlled Trial

**Published:** 2014-04

**Authors:** Marzieh Kargar, Zahra Jamshidi, Nooshin Beheshtipour, Narjes Pishva, Maryam Jamali

**Affiliations:** 1 Department of Pediatrics, School of Nursing and Midwifery, Shiraz University of Medical Sciences, Shiraz, Iran;; 2 Student Research Committee, School of Nursing and Midwifery, Shiraz University of Medical Sciences, Shiraz, Iran;; 3 Neonatology Center, Nemazee Hospital, School of Medicine, Shiraz University of Medical Sciences, Shiraz, Iran;; 4 Neonatology Center, Moslemin Hospital, School of Medicine, Shiraz University of Medical Sciences, Shiraz, Iran

**Keywords:** Head Covering, Phototherapy, Hypocalcaemia, Jaundice, Newborn

## Abstract

**Background:** Although phototherapy has been used for more than 60 years it has some complications. The light waves produced from phototherapy reduce melatonin concentration in newborns with subsequent hypocalcemia. We aimed to assess the effect of head covering on calcium and magnesium levels in full term newborns during phototherapy.

**Methods:** In this randomized controlled trial, 72 full-term icterus newborns weighing >2500 gr with indirect hyperbillirubinemia who received phototherapy at the phototherapy ward of Moslemin Hospital, Shiraz, southwest Iran, during March to September 2010 were recruited and divided randomly into two groups. The neonates in the case group received phototherapy while wearing a hat, while phototherapy was performed without hats for the control group. The newborns were in perfect health during the tests. The calcium and magnesium levels of each newborn were tested at baseline, and 24 hours after phototherapy and 48 after treatment. The variables were compared using Student’s *t*, Chi-square and repeat measurement tests.

**Results:** 14 (38.8%) newborns in the control group and 5 (13.8%) in the case group had hypocalcemia after phototherapy. A significant difference was found between the incidence of hypocalcemia in these two groups (P=0.03). However, magnesium levels did not change significantly (P>0.05).

**Conclusion:** Phototherapy caused hypocalcemia but it had no effect on magnesium levels. This can be prevented by covering the head during phototherapy with no need for prophylactic administration of calcium.

**Trial Registration Number: **IRCT2013102315134N1

## Introduction

According to the latest global reports and statistics, 60% of full-term newborns (Gestational age≥38 weeks) are afflicted with jaundice in the first week of their birth.^1^

In 1983, the actual effect of phototherapy on jaundice was proved by Black and his method has been used for icterus newborns until now.^2^ But this method also has some complications such as insensible water loss, mutation and DNA strand break, hyperthermia, tremor, retinal damage, bronze baby syndrome, and maternal-infant interaction.^3^

Hypocalcaemia has been reported as a reaction to phototherapy in premature and full term newborns.^4^^,^^5^ Some complications of hypocalcaemia in newborns are apnea, convulsion, muscle cramp, tremor, and tetanus.^6^ Hypocalcemia are causes long-term complications such as mental retardation, physical disability, and educational failure.^7^

Phototherapy leads to increased calcium absorption by the bones through radiating the pineal gland and reducing melatonin level.^5^ There is some evidence that the use of a stockinet cap to cover the head prevents phototherapy-induced hypocalcaemia.^8^

Some studies have suggested the administration of calcium for this condition, but intravenous calcium therapy may lead to bradycardia, cardiac arrest, and necrosis. On the other hand, monitoring intravenous infusions of calcium is difficult because of the shortage of trained staff.^4^

Hypocalcaemia and hypomagnesaemia may co-occur.^9^ The serum calcium level is regulated by magnesium via supplying the required hormones.^10^ We aimed to assess the effect of head covering on ionized serum calcium and magnesium levels in newborns with hyperbilirubinemia during phototherapy.

## Materials and Methods

**Figure 1:**
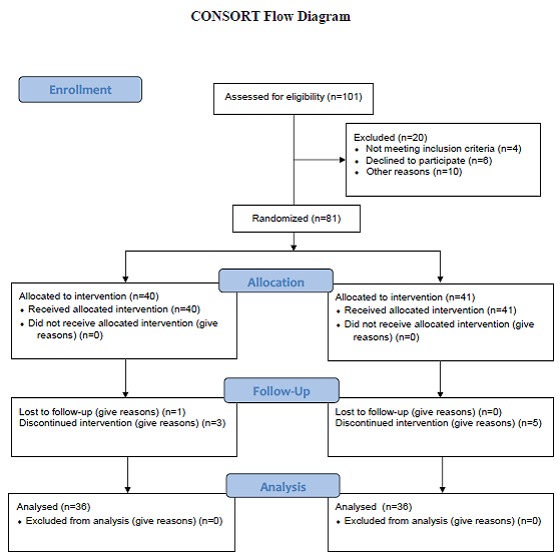
Consort flow diagram of the study participants

This randomized controlled trial was performed on 72 full-term healthy neonates with indirect hyperbilirubinemia who were admitted to phototherapy unit of Moslemin Hospital, Shiraz, southern Iran, from March to September 2010 (figure 1). Newborns that were under routine phototherapy were divided into two case and control groups, each including 36 infants, by random allocation. For systemic allocation the case and control groups were determined randomly using a coin at first. Then infants were placed into the case and then control group one by one. The protocol of the intervention was approved by the Ethics Committee of Shiraz University of Medical Sciences. Written informed consent was received from the parents.

We included full-term neonates who weighed more than 2500 grams and had normal serum calcium levels. Apart from icterus (indirect bilirubin between 13 to 18 mg/dl in full-term neonates) their physical status was completely normal. Sex, birth weight, weight at the time of phototherapy, indirect bilirubin levels, reticulocyte, hemoglobin and the type of feeding were recorded. Newborns with disorders such as asphyxia, hypothyroidism, G_6_PD, ABO/RH incompatibility, exchange transfusion, respiratory distress, sepsis, hemolytic anemia, and those who received IV therapy or their mothers had diabetes mellitus or thyroid disorders were excluded.

Routine phototherapy was used for the control group, and the occipital area of the neonates in the case group during phototherapy was covered by a hat. Calcium and magnesium levels were checked at baseline, 48 hours after starting phototherapy, and 24 hours after the treatment. All the infants were placed under phototherapy with 8 lamps, 40 watts, blue light with a wavelength of 470-410 nanometers, at a distance of 40-30 cm from the body surface area. A calcium level ≤7.5 mg/dl was considered as hypocalcaemia^11^ and Mg level ≤1.2 mg/dl was considered as hypomagnesaemia.^3^

Data were analyzed using Student’s t, Chi-square and repeat measurement tests. Statistical analysis was carried out using SPSS software, version 11.5. The sample size was calculated according to the following formula (α=0.05, β=0.2, σ=1.5, d=|x_1_–x_2_|=1/σ, z=1.96):

N=[2 (z_1-α/2_+z _1-β)_^2 σ^ ^2^]/d^2^

## Results

The mean±SD weight and age of the neonates were 3080±389.3 grams and 4.52±1.3 days, respectively. 32 (44.4%) neonates were boys and 40 (55.6%) were girls. The average time of phototherapy was three days (range: 3-5 days). The mean±SD serum bilirubin level was 14.3±1.2 mg/dl. Hypocalcaemia was found in 14 (38.88%) out of 36 newborns in the control group (table 1). The level of calcium in both groups is shown in the table 2.

**Table 1:** The comparison between control and study groups.

**Groups**	**Age (Day)**	**Sex** **(M/F)**	**Birth weight (gr)**	**Duration of phototherapy (Day)**	**Indirect bilirubin**	**Type of feeding**
Study	3.1±1.2	17/19	3130±490	3.5±0.8	14.5±1.1	
Control	3.7±1.6	15/21	3040±382	4.2±0.6	14.2±1.3
P value	<0.05	<0.05	<0.05	<0.05	<0.05	<0.05

**Table 2:** The frequency and comparison of calcium rate  in the control and study groups (48 hours after starting phototherapy)

**Ca level**	**Control**		**Study**		**P value**
	**Percent**	**No**	**Percent**	**No**	
<7.5	38.88%	14	13.88%	5	0.03
>7.5	62.11%	22	86.11%	31	
Total	100%	36	100%	36	

**Figure 2:**
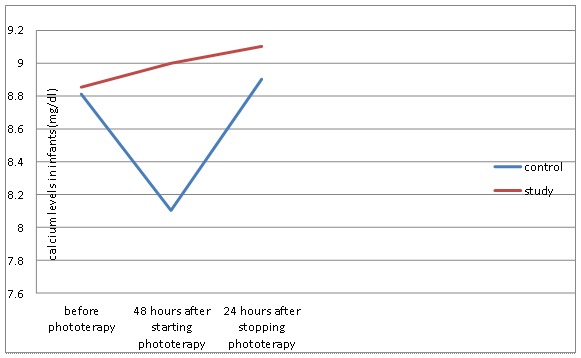
Comparison of calcium levels in infants in the control and study groups during phototherapy.

**Figure 3:**
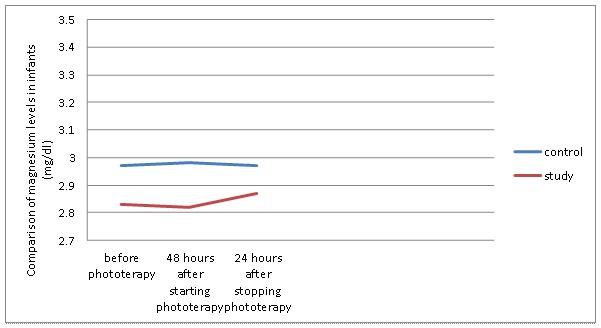
Comparasion of magnesium levels in infants in the control  and study  groups during phototherapy

There was a significant difference in the incidence of hypocalcaemia between the two groups (P=0.03, figure 2). Moreover, no newborn was affected by hypomagnesaemia during phototherapy (P=0.24, figure 3).

The calcium and magnesium levels at baseline, 48 hours after phototherapy, and 24 hours following phototherapy were compared using repeated measurements. (You have not mentioned the results of the repeated measurements at these three times) We found no significant difference between the type of delivery, feeding, sex, and age of the newborns and the prevalence of hypocalcaemia. 

## Discussion

Physiological jaundice is defined as indirect bilirubin increase of more than 12.9 mg/dl in full-term neonates.^11^ The efficacy of phototherapy in the prevention and treatment of hyperbilirubinemia in newborn infants has been well established. Phototherapy increases calcium absorption by the bones and leads to the reduction of melatonin levels which can be prevented by covering the occipital region.^5^ Changes in melatonin levels affect the incidence of hypocalcemia- induced phototherapy.^12^

Hypocalcaemia has been reported as a reaction to phototherapy in premature and full-term newborns. There are few studies evaluating the effect of phototherapy on serum calcium level with similar results.^4^ We found a significant difference in the incidence of hypocalcemia between the control group receiving routine phototherapy and the case group with covering occipital area by a hat during phototherapy.

We found that 14 (38%) newborns in the control group had hypocalcaemia (Ca≤7.5 mg/dl) 48 hours after phototherapy. Romagnoli and colleagues were the first to suggest an association between hypocalcaemia and phototherapy in neonates.^13^ Other researchers also observed a significant decrease in serum calcium levels in newborn rats after exposure to fluorescent day light.^5^ In another study on 20 term and 20 preterm neonates with hyperblirubinemia, 75% of term and 90% of preterm neonates developed hypocalcaemia after phototherapy.^14^ The results of the above mentioned studies are consistent with ours. In one study hypocalcaemia was observed in 30% of the term neonates receiving phototherapy,^15^ which is lower than the above mentioned studies. We found that three infants in the control group with calcium level below 7 mg/dl and were controlled by a physician.

The role of calcium in the body includes blood coagulation, neuromuscular excitability, cell membrane integrity and function, and cellular enzymatic and secretory activity. Cellular permeability to sodium ions and increased cell membrane excitability are the signs of hypocalcaemia and other non-specific signs are apnea, seizure, jitteriness, increased extensor tone, clonus, hyper-reflexia, and stridor.^16^

Studies on 63 full-term newborns weighting more than 2500 grams, confirmed the emergence of hypocalcaemia after phototherapy, and researchers recommended prophylactic calcium for one of the infants who developed apnea.^17^ In one study, 27.3% of the premature infants who were irritable had hypocalcaemia during phototherapy.^15^ Moreover, vitamin D had no role in the pathogenesis of phototherapy induced-hypocalcaemia^18^ and administration of calcium was thus recommended during phototherapy.^14^^,^^16^

Hypocalcaemia in infants may be symptomatic or asymptomatic.^19^ Some studies have reported asymptomatic hypocalcaemia in neonates.^4^^,^^11^ None of the newborns in our study had symptomatic hypocalcaemia and had phototherapy-induced hypocalcaemia.

In addition, sometimes hypomagnesaemia and hypocalcemia can occur concomitantly. One study reported that 16 out of 29 neonates developed concomitant hypomagnesaemia and hypocalcaemia.^9^ In case of decreased calcium levels, infants might also need magnesium.^3^ But in our study, no significant difference in magnesium levels was found between the two groups  48 hr after starting phototherapy (P=0.24), which indicated that none of the infants had magnesium amount below 1.2 mg/dl. It was not necessary to give magnesium to the infants with hypocalcaemia.

It is suggested that covering the pineal gland only helps prevent hypocalcaemia. The level of calcium in the control group reached the normal range after phototherapy. In fact, light affected melatonin concentration followed by a decrease in serum calcium level.  This study showed that neonates requiring phototherapy were at a higher risk of developing hypocalcaemia. 

## Conclusion

Head covering during phototherapy is an appropriate and safe method for preventing phototherapy-induced hypocalcaemia in icterus newborns. Using a hat can prevent hypocalcaemia; therefore, it is important to monitor calcium levels during phototherapy. It is suggested to carry out the same study for pre-term infants. 

## References

[1] Khosravi N, Samaie H, Mozhdehiazar K (2001). Percentage of serum albumin in full term newborns in Shahid Akbar Abadi Hospital. Razi Journal of Medical Sciences.

[2] Black S (1983). The bright side of phototherapy. MCN Am J Matern Child Nurs.

[3] Cloherty JP, Eichenwald EC, Stark AR (2008). Manual of neonatal care.

[4] Ehsanipoor F, Khosravi N, Jalali S (2008). The effect of hat on phototherapy -indused hypocalcemia in icteric newborns. Razi Journal of Medical Sciences.

[5] Hakanson DO, Bergstrom WH (1981). Phototherapy induced hypocalcemi a in newborn rats prevention by melatonin. Scienc.

[6] Velayati AA, Rahmati M, Khorgami MR, et al, Faranoosh M (2006). Nelson essentials of pediatrice.

[7] Pezeshki N, Mani KH (2005). Effect of calcium injection on prevalence of hypocalcemia in preterm newborns. Scientific Journal of Hamadan University of Medical Sciences & Health Services.

[8] Whaley L, Wong D (2004). Text book of infant and pediatric nursing care.

[9] Bahjati SH, Anjarani S, Rastgar H (2003). Hypocalcemia & other factors effects on it. Iranian Journal of Pediatrics.

[10] Smeltzer SC, Bare BG, Hinkle JL, Salemi s (2004). Brunner & suddarths textbook of medical surjical nursing.

[11] Karamifar H, Pishva N, Amirhakimi GH (2002). Prevalence of phototherapy induced hypocalcemia. Iranian Journal of Medical Sciences.

[12] Cardinali DP, Ladizesky MG, Boggio V (2003). Melatonin effects on bone: experimental facts and clinical perspectives. Journal of Pineal Research.

[13] Romagnoli C, Polidori G, Cataldi L (1979). Phototherapy induced hypocalcaemia. Journal of Pediatrics.

[14] Sethi H, Saili A, Dutia AK (1993). Phototherapy induced hypocalcemia. Indian Pediatr.

[15] Jain BK, Singh H, Singh D, Toor NS (1998). Phototherapy induced hypocalcemia. Indian Pediatr.

[16] Yadav RK, Sethi RS, Sethi AS (2012). The Evaluation of Effect of Phototherapy on Serum Calcium Level. People’s Journal of Scientific Research.

[17] Eghbalian F, Monsef A (2002). Phototherapy-induced hypocalcemia in icteric newborn. Iran J Med Sci.

[18] Zecca E, Romagnoli C, Tortol G (1983). In effective of vitamin 25 (OH) D3 in the prevention of hypocalcemia induced by phototherapy. Pediatr Med Chir.

[19] Greenbaum LA, Behrman RE, Kliegman RM, Jenson HB (2004). Pathophysiology of body fluids and Fluid therapy. Textbook of Pediatrics.

